# Gastric and intestinal barrier impairment in tropical enteropathy and HIV: limited impact of micronutrient supplementation during a randomised controlled trial

**DOI:** 10.1186/1471-230X-10-72

**Published:** 2010-07-06

**Authors:** Paul Kelly, Tamara Shawa, Stayner Mwanamakondo, Rose Soko, Geoff Smith, G Robin Barclay, Ian R Sanderson

**Affiliations:** 1 Blizard Institute of Cell and Molecular Science, Barts & The London School of Medicine, Queen Mary University of London, London, UK; 2 Tropical Gastroenterology and Nutrition group, University of Zambia School of Medicine, Lusaka, Zambia; 3 London School of Hygiene and Tropical Medicine, London, UK; 4 Imperial College School of Medicine, Charing Cross Hospital, London, UK; 5 Scottish National Blood Transfusion Service Cell Therapy Group, MRC Centre for Regenerative Medicine, University of Edinburgh, Edinburgh, UK

## Abstract

**Background:**

Although micronutrient supplementation can reduce morbidity and mortality due to diarrhoea, nutritional influences on intestinal host defence are poorly understood. We tested the hypothesis that micronutrient supplementation can enhance barrier function of the gut.

**Methods:**

We carried out two sub-studies nested within a randomised, double-blind placebo-controlled trial of daily micronutrient supplementation in an urban community in Lusaka, Zambia. In the first sub-study, gastric pH was measured in 203 participants. In the second sub-study, mucosal permeability, lipopolysaccharide (LPS) and anti-LPS antibodies, and serum soluble tumour necrosis factor receptor p55 (sTNFR55) concentrations were measured in 87 participants. Up to three stool samples were also analysed microbiologically for detection of asymptomatic intestinal infection. Gastric histology was subsequently analysed in a third subset (n = 37) to assist in interpretation of the pH data. Informed consent was obtained from all participants after a three-stage information and consent process.

**Results:**

Hypochlorhydria (fasting gastric pH > 4.0) was present in 75 (37%) of participants. In multivariate analysis, HIV infection (OR 4.1; 95%CI 2.2-7.8; *P *< 0.001) was associated with hypochlorhydria, but taking anti-retroviral treatment (OR 0.16; 0.04-0.67; *P *= 0.01) and allocation to micronutrient supplementation (OR 0.53; 0.28-0.99; *P *< 0.05) were protective. Hypochlorhydria was associated with increased risk of salmonellosis. Mild (grade 1) gastric atrophy was found in 5 participants, irrespective of *Helicobacter pylori *or HIV status. Intestinal permeability, LPS concentrations in serum, anti-LPS IgG, and sTNFR55 concentrations did not differ significantly between micronutrient and placebo groups. Anti-LPS IgM was reduced in the micronutrient recipients (*P <*0.05).

**Conclusions:**

We found evidence of a specific effect of HIV on gastric pH which was readily reversed by anti-retroviral therapy and not mediated by gastric atrophy. Micronutrients had a modest impact on gastric pH and one marker of bacterial translocation.

**Trial Registration:**

Current Controlled Trials ISRCTN31173864

## Background

Defence against infectious disease is a matter of the highest importance for the health and development of people living in tropical regions. Diarrhoeal disease is a major contributor to infectious disease morbidity and mortality in developing countries especially in children and in AIDS patients [1,2]. Malnutrition has long been associated with increased susceptibility to, and worse outcomes from, infectious disease. In terms of diarrhoeal disease micronutrients may be critical for robust host defence. In particular, deficiency of vitamin A is associated with increased mortality in children and the benefit of vitamin A supplementation is probably at least partly attributable to reduced mortality from diarrhoea [3]. There is convincing evidence that zinc supplementation improves outcomes in children with diarrhoea [4] and most trials suggest that it reduces diarrhoea incidence when given prophylactically [5,6]. In HIV infection there is mixed evidence that micronutrient supplementation reduces morbidity and mortality [7-9], and some evidence suggests that selenium supplementation can raise CD4 counts in AIDS patients [10,11]. However, the mechanisms by which micronutrients reduce the impact of diarrhoeal disease are entirely unknown. Evidence that nutrition directly controls cell-mediated immunity has been largely discredited [12], and there is no evidence that nutritional interventions have an impact on humoral immunity [13]. In view of the fact that vitamin A deficiency is associated with breaches of epithelial barriers [14] and zinc deficiency is associated with disorders of Paneth and other intestinal epithelial cells [15], we hypothesised that micronutrient affects barrier function in the gut.

Bacterial translocation from the gut plays a major role in infection, disease progression and mortality in cirrhosis [16-18], in hepatitis C [19], and in systemic sepsis syndrome [17]. Kupffer cells in the liver constitute the largest compartment of macrophages in the body, presumably there in order to deal with translocation, and there are physico-chemical barriers to penetration and translocation which include gastric acid, the mucus layer, and the integrity of the epithelial layer [20,21]. Gastrointestinal barrier function therefore represents the sum of several factors, and can only loosely be defined. At least one component of this barrier, gastric acid (both in terms of resting pH and pentagastrin-stimulated acid output) has been known for many years to be impaired in AIDS [22,23].

It has been known for many years that HIV infection leads to a severe T cell depletion in the intestinal mucosa [24,25]. Recent data indicate that, at least in the SIV model, this T cell population never recovers completely [26] and it has been proposed that the enteropathy observed in HIV infection leads to bacterial translocation, driving HIV disease progression [27]. We and others, however, have shown that HIV-related enteropathy is largely associated with the later stages of HIV infection [28-34]. It seems likely that any intervention which could ameliorate or reverse impaired barrier function in HIV infected patients might reduce the systemic immune activation. This immune activation is reflected in elevated circulating cytokines such as interferon -γ and tumour necrosis factor-α (TNF) which behave as predictors of mortality in Zambian adults with AIDS [35]. As part of a randomised, placebo-controlled trial of a daily multiple micronutrient supplement [36], we studied several facets of intestinal immunity and here we report our analysis of gastric pH, intestinal permeability, LPS and TNF concentrations in serum.

## Methods

Patients were studied during the course of a cluster-randomised, placebo-controlled trial of daily micronutrients supplementation in a community in Lusaka, Zambia. The trial showed no evidence of benefit on diarrhoea incidence or CD4 count, but severe episodes of diarrhoea and mortality in HIV infection did appear to be reduced [36]. In summary, 500 adults with or without HIV infection living in one section of Misisi, Lusaka, were recruited and cluster-randomised by household. The multiple micronutrient (MM) supplement was designed to provide at least the recommended nutrient intake [37] of 15 nutrients (Table 1), and participants were crossed over to the opposite treatment allocation after 2 years. The data were collected at a time when highly active anti-retroviral treatment (HAART) was being introduced into Zambia so some participants had been taking HAART for varying periods of up to 15 months. Ethical approval was obtained from the Research Ethics Committees of the University of Zambia and the London School of Hygiene and Tropical Medicine. The trial was registered as ISRCTN31173864.

**Table 1:** Composition of the micronutrient tablet

Micronutrient	Amount	**RNI**^**37**^
β-carotene	4.8 mg	4.2 mg equivalent

Ascorbic acid (vitamin C)	70 mg	40 mg

Cholecalciferol (vitamin D_3_)	5 μg	-

Tocopherol (vitamin E)	10 mg	4 mg (uncertain)

Thiamine (vitamin B_1_)	1.4 mg	1.0 mg

Riboflavin (vitamin B_2_)	1.4 mg	1.3 mg

Niacin	18 mg	17 mg

Pyridoxine (vitamin B_6_)	1.9 mg	1.4 mg

Cyanocobalamin (vitamin B_12_)	2.6 μg	1.5 μg

Folic acid	400 μg	200 μg

Iron	30 mg	14.8 mg (women), 8.7 mg (men)

Zinc	15 mg	9.5 mg

Copper	2 mg	1.2 mg

Selenium	65 μg	75 μg

Iodine	150 μg	140 μg

Composition of the trial medication (one tablet was given each day) compared to the UK recommended nutrient intake (RNI). The RNI for vitamin D in healthy adults exposed to sunlight is zero if appropriate lipid precursors are present in the diet [37].

The current study was nested within the main trial, which was conducted from July 2003 to December 2006. Two sub-studies were carried out on different groups of participants identified at random during the course of the main trial: (i) measurement of gastric pH (n = 203) in participants who had been taking MM or placebo for between 4 and 19 months (median 9 months, IQR 7-15 months); (ii) studies of intestinal permeability (using four sugars), translocation (using LPS and anti-LPS antibodies) and immune activation (using TNF receptor (TNFR) p55) in 86 participants. The gastric pH and translocation sub-studies were carried out at different times for logistic reasons (i.e. workload), the pH study after the cross-over and the translocation study before the cross-over, so although permeability, LPS and TNF were assayed in the same individuals on the same day, no before/after cross-over comparisons within the same individual are available. The allocation of participants to the sub-studies was largely at random, but the translocation study did include 18 participants who elected to undergo the permeability testing rather than the endoscopy. As the treatment allocation was carefully randomised and fully double-blind, we anticipated that this would have no influence on the results of the comparisons within each sub-study. Stool samples (up to 3) were collected from each participant for analysis of carriage of salmonellae and shigellae using standard techniques.

### Gastric pH measurements

To collect gastric acid, endoscopy was carried out in fasting participants under sedation with midazolam. Aspirates were collected by attaching a 10 ml syringe to the biopsy port of a Pentax FG29W gastroscope which had been carefully flushed with clean water and then air prior to introduction. No aspiration was carried out until the tip of the scope was in the pool of intra-gastric fluid. Gastric pH was measured in aspirates of intra-gastric fluid using Macherey-Nagel pH test strips which are accurate to pH changes of 0.5 (D-52348, Macherey-Nagel, Germany) to avoid contamination of pH probes.

### Permeability: four sugar absorption/secretion testing

The four sugar test solution used contained 0.5 g xylose, 1 g rhamnose, 5 g lactulose and 0.2 g 3-O-methyl D-glucose (Sigma chemicals, Poole, Dorset, UK) in 100 ml distilled water to constitute an approximately isosmolar solution. After collecting a pre-test sample to exclude prior ingestion of test sugars, participants drank this solution at time 0, and then urine samples were collected for exactly 5 hours and preserved with merthiolate (approximately 0.04% final concentration). Urine samples were frozen at -80°C for transport and analysed by thin layer chromatography as previously described [28,38]. Xylose recovery, Rhamnose: 3-O-methyl D-Glucose (R:G) ratio, and Lactulose: Rhamnose (L:R) ratio were analysed.

### Lipopolysaccharide concentrations in serum

Lipopolysaccharide (LPS) and anti-lipopolysaccharide antibodies (IgG and IgM) were measured as markers of bacterial translocation. LPS concentrations in serum were measured using the Pyrochrome-LAL kit (Associates of Cape Cod Inc, East Falmouth, MA) according to the manufacturer's instructions and optimised for incubation time which was determined to be 60 minutes. Endotoxin core antibodies (EndoCAb) IgM and IgG were measured by ELISA as previously described [39,40]. In brief, polystyrene microplates were pre-coated with an equimolar mixture of incomplete-core rough-mutant endotoxins from each of four species of Gram-negative bacteria, complexed with polymyxin B. An eight-point standard curve was constructed using doubling dilutions of a pooled serum calibrated in EndoCAb median units, where 100 (%) was the median value for IgG or IgM in 1,000 healthy Scottish adults. Test and control samples were diluted 1:200 with dilution buffer and 100 μL of each sample added in triplicate to the plate and incubated for 1 h at 37C. After washing three times with wash buffer (Dulbecco phosphate buffered saline containing 0.1% [v/v] Tween 20), 100 μL of a diluted alkaline phosphatase conjugated goat antihuman IgG or IgM antibody was added to each well. After incubation for 1 h at 37C the plates were washed three times with wash buffer then once with distilled water and blotted dry. Substrate (100 μL per well), comprising 1 mg/ml disodium p-nitrophenylphosphate dissolved in 1 M diethanolamine buffer with 0.5 mM magnesium chloride, was added and the plate incubated at room temperature in the dark for 20-30 min. The reaction was stopped with 50 μL per well of 2 M sodium hydroxide and read at 405 nm.

### Tumour necrosis factor receptor p55 concentrations

In order to look for correlation between LPS or anti-LPS antibodies and macrophage activation, we measured TNFR (p55) in the same sera which were assayed for LPS and anti-LPS. TNFR was measured by ELISA (R&D systems, Abingdon, UK) according to the manufacturer's instructions.

### Assessment of gastric histology

When it became apparent that HIV was associated with hypochlorhydria, we submitted for histological examination gastric biopsies from antrum and fundus from 10 HIV infected and 27 uninfected participants from the same study cohort obtained during an earlier study of *Helicobacter pylori *and HIV [41]. Of these, 3 had CD4 counts under 200 cells/μl, all of whom were investigated prior to receiving treatment with anti-retroviral drugs. Haematoxylin and eosin-stained sections were assessed using the modified Sydney system for evaluating gastric changes related to *Helicobacter pylori *[42]. Extent of intestinal metaplasia was assessed by staining with Alcian blue - Periodic Acid Schiff's (AB-PAS). Density of *Helicobacter pylori *staining was assessed using a modified Giemsa stain [43]. The Modified Sydney System utilises a 0-3 range score across 4 domains as previously described. For atrophy the score ranges from 0 (normal gland structure), 1 (minimal gland loss), 2 (moderate gland loss or replacement with a chronic inflammatory infiltrate) and 3 (severe gland loss/few glands seen).

### Data analysis

Hypochlorhydria was defined as fasting gastric pH > 4.0. Variables are presented as median and interquartile range (IQR) and non-parametric statistics were used (Kruskal-Wallis test for comparison of two medians, and correlation coefficients were calculated on log-transformed data). Statistical analysis was carried out using STATA 10 (Stata Corp, College Station, Texas).

Sample size calculations were performed *post hoc *as this was a sub-group analysis of a clinical trial with diarrhoea as the primary endpoint [36]. Reference to published work [44] indicates that LPS concentrations in AIDS patients might be expected around 60 pg/ml and in controls around 30 pg/ml. To detect a difference between these means, with standard deviation of 44 pg/ml as observed here and with 80% power at 95% confidence, would require 34 in each group.

## Results

The assessments of barrier function reported here were performed on subsets of the larger trial group, all of whom were randomly allocated to micronutrient or placebo supplementation in a double-blind manner; the demographic characteristics of these subsets are shown in Table 2.

**Table 2:** Demographic and clinical characteristics of study groups for the analysis of gastric acid and the analysis of permeability, translocation and immune activation

	Gastric acid sub-group		Translocation sub-group	
	MM	Placebo	MM	Placebo
	(n = 100)	(n = 103)	(n = 38)	(n = 49)
Age (yrs) (median, 5^th^/95^th ^centiles)	31 (19-65)	36 (20-62)	41 (19-56)	29 (18-51)
Sex Male	35	48	8	14
Female	65	55	30	35
BMI (kg/m^2^) (median, 5^th^/95^th ^centiles)	21.3 (17.9-31.7)	21.9 (17.7-31.6)	22.1 (18.3-36.3)	23.2 (18.0-41.6)
MUAC (cm) (median, 5^th^/95^th ^centiles)	28.0 (24.0-38.0)	27.4 (23.0-34.3)	26.0 (22.5-37.0)	26.8 (21.9-38.2)
HIV positive	40 of 97 tested	44 of 101 tested	5 of 29 tested	12 of 46 tested
CD4 count (median, 5^th^/95^th ^centiles)	382 (68-716)	349 (161-659)	362 (331-634)	466 (190-936)
CD4 count < 200 cells/μL	7/40	4/44	0/5	2/12

*P *values are not shown: none of the comparisons between MM and placebo were significant

### Gastric pH was reduced at all stages of HIV infection

**Figure 1:**
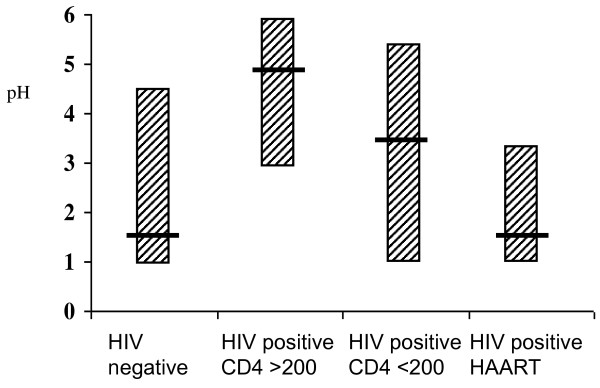
**Gastric pH in relation to HIV status and anti-retroviral therapy**. The difference across all groups was significantly different using the Kruskal-Wallis test (*P *= 0.0005).

In 203 participants in whom pH was measured in fasting gastric aspirates, the median pH was 3 (IQR 1-5.5). Hypochlorhydria was present in 75 (37%). Of the 72 participants with hypochlorhydria whose HIV status was known, 42 were HIV seropositive (OR 3.1, 95%CI 1.6-5.9; *P *= 0.0003). Anti-retrovirals were being taken by 14 participants at the time of the analysis, so the relationship between gastric pH and HIV status was further assessed by breaking down HIV infected participants into three groups: HIV positive with high CD4 count (>200 cells/μl), HIV positive with low CD4 count (200 cells/μl or less), and HIV positive taking HAART (all except one of whom had CD4 counts < 200 cells/μl when initiated). HIV infection was associated with hypochlorhydria, but not in patients taking HAART (Figure 1). The proportion of participants with hypochlorhydria was 29/114 (25%) in the HIV negative group, 36/62 (58%) in the HIV positive group with higher CD4 counts, 4/8 (50%) in the HIV positive group with low CD4 count, and 3/14 (21%) in the group taking anti-retrovirals. Intestinal infection with two species of pathogenic bacteria was more likely in hypochlorhydric patients: *Salmonella enterica *serovar typhimurium and/or *Shigella *spp. infections were found in 11/198 (5%) of samples from participants with hypochlorhydria compared with 7/357 (2%) of samples from those without (*P *< 0.05).

### Gastric pH was decreased by MM supplementation in multivariate analysis

Participants had been taking MM or placebo for a median of 9 (IQR 7-15) months, but the duration of consumption of trial medication had no effect on pH. Median (IQR) pH was 2.3 (1-5) in 100 participants allocated to MM and 3 (1-5.5) in 103 allocated to placebo (*P *= 0.31). Hypochlorhydria was found in 32 (32%) participants on MM and 43 (42%) on placebo (*P *= 0.19). Multivariate analysis was carried out using logistic regression with hypochlorhydria as the dependent variable, and including age, sex, low body mass index, HIV infection, low CD4 count, taking HAART, and treatment allocation. In the final model, HIV infection (OR 4.1, 95%CI 2.2-7.8; *P *< 0.001) was associated with hypochlorhydria; taking HAART (OR 0.16, 0.04-0.67; *P *= 0.01) and allocation to micronutrient supplementation (OR 0.53, 0.28-0.99; *P *< 0.05) were protective against it. Low BMI (18.5 kg/m^2 ^or less) was not predictive of hypochlorhydria and did not explain the association between HIV and hypochlorhydria.

### Intestinal permeability was not reduced by MM supplementation

Four sugar testing and fractional urine recovery over 5 hours was used to obtain measurements of Xylose absorption, Rhamnose:3-O-methyl D-Glucose absorption ratio, and Lactulose:Rhamnose permeation ratio in a subset of 87 participants drawn at random from the larger group. Of these participants, only one had a CD4 count below 200 cells/μl and none were taking anti-retroviral drugs. These three measures of intestinal function and permeability did not differ in MM compared to placebo groups (Table 3). Neither was there any significant difference between HIV groups (Table 3).

**Table 3:** Measures of intestinal permeability, absorptive capacity, bacterial translocation, and tumour necrosis factor receptor p55, analysed both by treatment allocation and by HIV status

Measure	By treatment allocation			By HIV status*		
	
	MM	Placebo	*P*	HIV positive	HIV negative	*P*
	(n = 37)	(n = 49)		(n = 17)	(n = 57)	
Xylose (%)	21.4 (18.9-27.4)	21.2 (16.3-26.4)	0.49	20.3 (17.9-23.7)	21.2 (15.4-27.4)	0.94

R:G	0.35 (0.32-0.40)	0.38 (0.34-0.42)	0.23	0.39 (0.35-0.43)	0.36 (0.32-0.41)	0.12

L:R	0.057 (0.045-0.088)	0.048 (0.036-0.071)	0.12	0.059 (0.036-0.071)	0.050 (0.036-0.077)	0.49

LPS (pg/ml)	50.7 (24.8-115.1)	51.6 (22.0-107.9)	0.52	64.7 (29-113)	51.6 (20-109)	0.56

anti-LPS IgM (units)	95 (58-148)	127 (100-175)	0.047	117 (89-134)	118 (66-190)	0.46

anti-LPS IgG (units)	115 (63-201)	152 (64-272)	0.60	142 (98-293)	153 (63-223)	0.33

TNFp55 (pg/ml)	1.13 (1.01-1.40)	1.22 (1.02-1.40)	0.62	1.32 (0.99-1.64)	1.14 (1.04-1.38)	0.37

* HIV testing was declined by 12 participants. The units given for anti-LPS antibodies are in reference to Scottish blood donor median value (see Methods).

### Anti-LPS IgM was reduced by MM supplementation

LPS was assayed in sera from the same 87 participants undergoing permeability testing as above. LPS concentrations did not differ in MM and placebo groups, nor was there any difference between HIV seropositive and HIV seronegative participants (Table 3). No such differences were observed in anti-LPS IgG in serum either. However, anti-LPS IgM was significantly reduced in micronutrient recipients (Table 3). No significant correlation was found between LPS or anti-LPS levels in serum and the gut absorption or permeability markers, with the exception of log-transformed xylose recovery which was negatively correlated with log-transformed anti-LPS IgG (*r *= -0.30; *P *= 0.006).

### TNFR was not reduced by MM but was correlated with anti-LPS IgM

**Figure 2:**
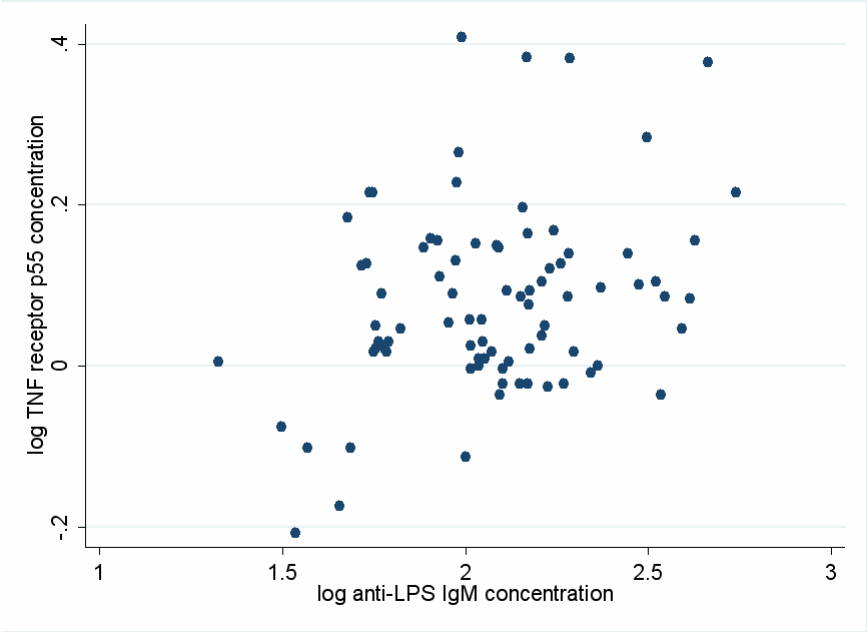
**Correlation between serum concentrations of TNFR55 and anti-LPS IgM**. The correlation coefficient (*r *= 0.30, *P *= 0.006) was derived from log-transformed data.

TNFR was measured as receptor p55, again in the same sera in which LPS and anti-LPS antibodies were assayed. No difference was observed either by treatment allocation or by HIV status (Table 3). The correlation between measures of translocation and TNFRp55 was also examined (Figure 2). The correlation coefficient between log-transformed TNFRp55 and anti-LPS IgM was significant (*r *= 0.30, *P *= 0.006), as was the correlation between TNFRp55 and anti-LPS IgG (*r *= 0.27, *P *= 0.01). Using multiple linear regression to define which immunoglobulin class was more strongly correlated with TNF activation, the IgM was significant associated with TNFRp55 while the IgG was not.

### HIV-related hypochlorhydria is not due to gastric atrophy

**Figure 3:**
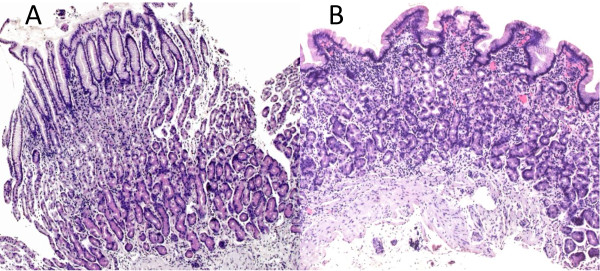
**Gastric histology**. Representative histological sections from gastric biopsies from a patient with (A) normal gastric histology and (B) the most severe gastric atrophy observed, which was classified as mild. No moderate or severe atrophy was seen.

In order to examine the mechanism of the hypochlorhydria, histological examination of antral and fundal gastric tissue was performed on 37 patients. No evidence of intestinal metaplasia was found, even though all except 4 (3 HIV seropositive, 1 HIV seronegative) had positive serology for *H. pylori*, and *Helicobacter*-like organisms were visible in 30 out of the 37 histological sections (density recorded as low in 8, intermediate in 14, and high in 8). All Helicobacter-infected gastric mucosa showed some evidence of gastritis. Gastric atrophy of mild grade was observed in biopsies from 5 participants (3 HIV seropositive, 2 HIV seronegative; *P *= 0.11), all of whom were seropositive for *H. pylori*. No moderate or severe gastric atrophy was observed (Figure 3).

## Discussion and Conclusions

In this study we examined several components of the gastrointestinal barrier against translocation of bacteria and their products, in relation to HIV status and to micronutrient supplementation. We found that hypochlorhydria in fasted adults was associated with HIV infection irrespective of stage, reversed by anti-retroviral therapy and, when adjusted for HIV status in multivariate analysis, reduced by micronutrient supplementation. Although a large body of work has defined hypochlorhydria in terms of gastric acid secretion rate, in this study we only examined gastric pH in one fasting aspirate obtained by endoscopy. While our single fasting sample is almost certainly not as sensitive in detecting hypochlorhydria as measurement of acid secretion rate would be, it is relevant to infectious disease as a snapshot of gastric pH represents the chemical barrier encountered by a pathogen on ingestion. Impaired gastric pH was associated with asymptomatic colonisation by two potentially pathogenic bacterial species, confirming the relevance of this way of analysing gastric pH for this situation.

We found little evidence that micronutrient supplementation reduced markers of intestinal permeability or bacterial translocation, except for a reduction in anti-LPS IgM concentrations in serum which just reached statistical significance. Interestingly, the anti-LPS IgM was clearly correlated with TNF pathway activation, implying that translocation is indeed a determinant of systemic immune activation in the mixed HIV/tropical enteropathy present in this population. Although anti-LPS IgM was reduced in the MM group, TNFR was not, and it may be that a larger reduction in translocation is required before an effect on TNF pathway activation is detectable. We previously noted [36] that our intervention had only modest effect on blood measures of vitamin A and zinc, even though the erythrocyte folate response was good, and we speculate that higher doses of micronutrients may have an enhanced effect.

We do not yet have an explanation for our findings that HIV infection is associated with hypochlorhydria. We had speculated prior to this study that hypochlorhydria would be attributable to malnutrition (low BMI) but this was not the case. Other workers have previously reported changes in parietal cell morphology [22] but we found minimal evidence of gastric atrophy and we propose that it is due to a specific effect of HIV itself, or perhaps through cytokine secretion, on the parietal cell. We have previously shown that HIV infection is associated with reduced peptic ulceration in Zambian adults co-infected with *Helicobacter pylori *infection [41], which may well be explained by the hypochlorhydria we report here, but the cellular and molecular nature of the interaction between HIV and *H. pylori *needs further work. Unlike increased permeability, hypochlorhydria seems to occur at any stage of HIV disease.

In addition to the effect of hypochlorhydria on intestinal infection reported here, and an effect on bacterial overgrowth previously reported [23], it might be expected to lead to impaired nutrient absorption, especially iron which is preferentially absorbed in the Fe^2+ ^form. Gastric acid converts Fe^3+ ^to Fe^2+^. Iron deficiency in children has been reversed by *H. pylori *eradication [45,46], although in another study there was no difference in fractional absorption of iron from a test meal in hypochlorhydric children [47]. The impact of HIV-related hypochlorhydria on iron metabolism remains to be established.

Recent data [27,44] have given fresh support to the hypothesis that in HIV infection increased intestinal permeability is responsible for bacterial translocation and systemic immune activation. Although we found a significant correlation between anti-LPS IgM (a marker of bacterial translocation) and TNFRp55, we found no correlation between permeability and translocation markers. One possible explanation for this is that background tropical enteropathy [28,38] allows translocation in the absence of HIV, perhaps by mechanisms other than paracellular permeability. The median LPS concentration in serum in the HIV negative participants in our current study (51 pg/ml) was similar to that found in chronic HIV infection in the American study and much higher than the HIV seronegative participants in that study (approximately 30 pg/ml) [44]. Alternatively, it may be that lactulose permeation is not an appropriate measure of permeability for assessing risk of bacterial translocation, as it reflects a much smaller pore size than would be required to permit translocation of bacteria or even LPS. Lactulose is an imperfect marker of small intestinal 'leakiness' but in the absence of non-invasive alternatives it has been widely used [38]. There are no widely used markers of colonic 'leakiness', which is a major weakness when it comes to understanding translocation. A third explanation is that bacterial translocation due to increased intestinal permeability is mainly a feature of advanced HIV infection. In a previous study of intestinal permeability we found that permeability was only elevated in HIV infected adults with low CD4 counts [28]. Only one of the participants we assessed here had a CD4 count below 200 cells/μL. Previous studies, with few exceptions [48,49], mostly show that HIV enteropathy is associated with late-stage disease [28-34]. There are few data from populations where tropical enteropathy is present, but our previous data from Zambia and older data from Uganda [50] support the idea that there is little difference in permeability between early HIV and tropical enteropathy.

## Competing interests

The authors declare that they have no competing interests.

## Authors' contributions

The study was designed by PK and IRS. Data collection and interaction with participants was carried out by PK, SM and RS. TS carried out most of the laboratory assays, guided by PK and GRB. GS carried out the histological analysis. The manuscript was written and revised by PK with discussion from all authors. All authors read and approved the final manuscript.

## Pre-publication history

The pre-publication history for this paper can be accessed here:

http://www.biomedcentral.com/1471-230X/10/72/prepub
